# Research progress of drug eluting balloon in arterial circulatory system

**DOI:** 10.3389/fcvm.2024.1287852

**Published:** 2024-03-20

**Authors:** Keji Lu, Xianglin Ye, Yaoxuan Chen, Peng Wang, Meiting Gong, Bing Xuan, Zhaobing Tang, Meiling Li, Jun Hou, Ke Peng, Haifeng Pei

**Affiliations:** ^1^ Department of Cardiology, The Affiliated Hospital, Southwest Medical University, Luzhou, China; ^2^ School of Medical and Life Sciences, Chengdu University of TCM, Chengdu, China; ^3^ Department of Cardiology, The General Hospital of Western Theater Command, Chengdu, China; ^4^ Department of Rehabilitation, The General Hospital of Western Theater Command, Chengdu, China; ^5^ Department of Cardiology, Chengdu Third People's Hospital, Chengdu, China

**Keywords:** drug eluting balloon, arterial circulatory system diseases, coronary artery diseases, instent restenosis, paclitaxel

## Abstract

The arterial circulatory system diseases are common in clinical practice, and their treatment options have been of great interest due to their high morbidity and mortality. Drug-eluting balloons, as a new type of endovascular interventional treatment option, can avoid the long-term implantation of metal stents and is a new type of angioplasty without stents, so drug-eluting balloons have better therapeutic effects in some arterial circulatory diseases and have been initially used in clinical practice. In this review, we first describe the development, process, and mechanism of drug-eluting balloons. Then we summarize the current studies on the application of drug-eluting balloons in coronary artery lesions, in-stent restenosis, and peripheral vascular disease. As well as the technical difficulties and complications in the application of drug-eluting balloons and possible management options, in order to provide ideas and help for future in-depth studies and provide new strategies for the treatment of more arterial system diseases.

## Graphical Abstract

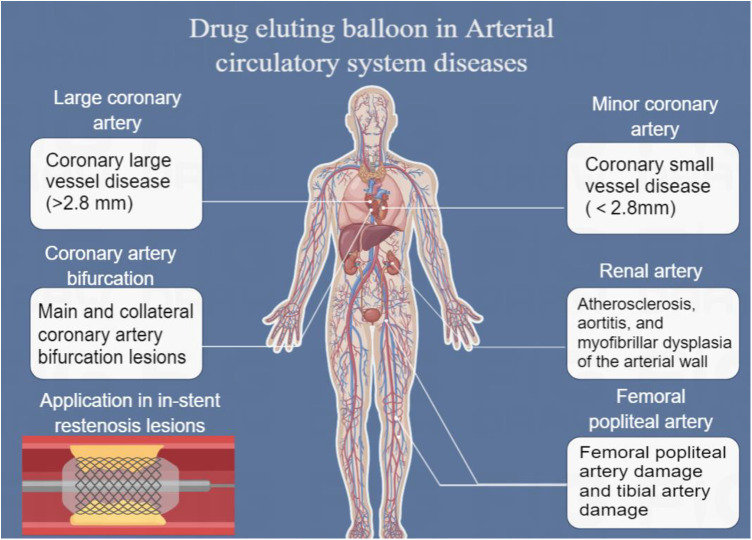

## Highlights

1. We describe the development, process, and mechanism of drug-eluting balloons.
2. We summarize the current studies on the application of drug-eluting balloons in coronary artery lesions, in-stent restenosis and peripheral vascular disease.
3. We have summarized numerous clinical trials and demonstrated the safety and effectiveness of drug-eluting balloons in the treatment of diseases including coronary heart disease and lower extremity vascular disease: ① For small vessel, large vessel and bifurcation lesions of coronary artery, their short term efficacy is favorable and safety is high, while their long term efficacy for large vessel lesions of coronary artery is also excellent ② For femoral and popliteal arteries, their performance is also sound ③ For renal blood vessels, children's cardiac vessels and in-stent restenosis, there have been studies or reports proving their treatment feasibility, but a large number of studies are needed to prove their safety and efficacy.
4. By reviewing the progress of drug-eluting balloon research mentioned above, we can provide new ideas for the treatment of more arterial system diseases.

**Significance Statement:** This review can provide ideas and help for future in-depth studies and provide new strategies for the treatment of more arterial system diseases.

## 1 Drug eluting balloon overview

New stenosis and restenosis of blood vessels are frequent causes of cardiovascular disease, and angioplasty is one of the main strategies to treat such diseases (1, 2). Drug-eluting balloon (DEB) is a new angioplasty technique which combines balloon angioplasty and drug-eluting technology. It uses a balloon catheter as a delivery medium to deliver drugs that inhibit cell proliferation to the lesion site to achieve vasodilatation and inhibit endothelial cell proliferation at the same time. Because of its good therapeutic effect in preventing intimal proliferation and restenosis, it is increasingly favored by multidisciplinary researchers.

### 1.1 Traceability of drug eluting balloon

**Figure 1:**
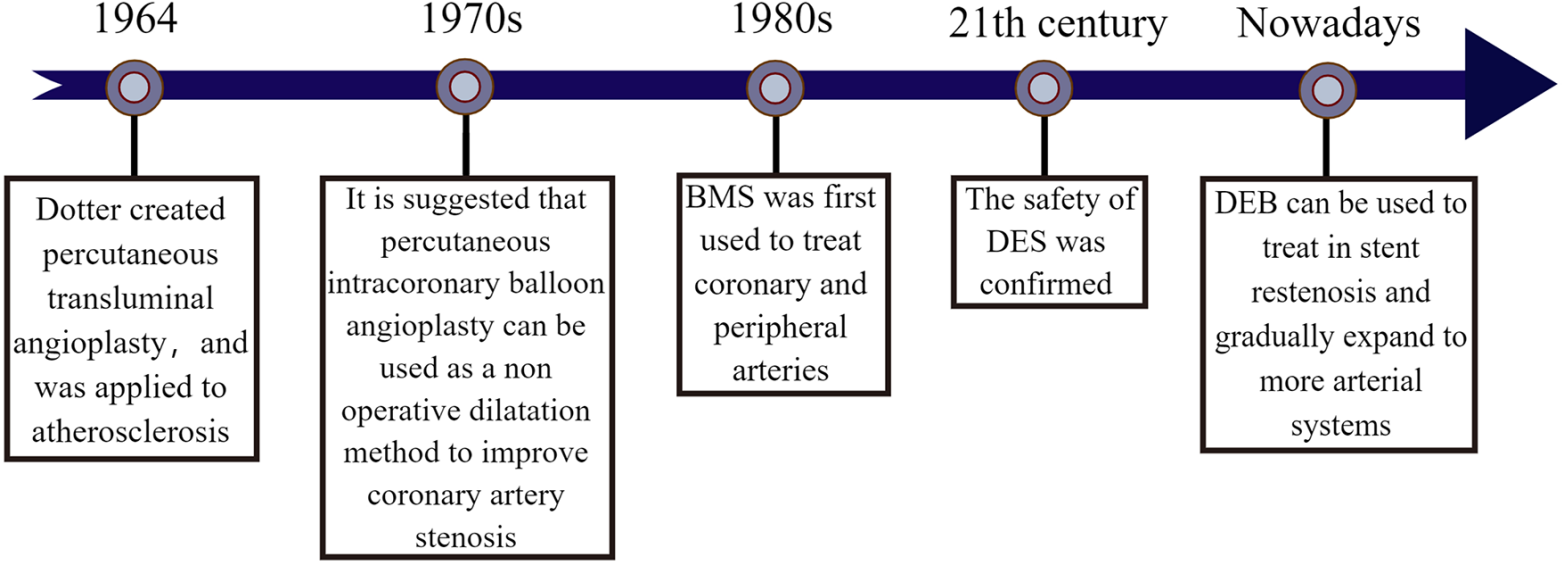
The origin of drug balloon. The figure illustrates the creation of percutaneous transluminal angioplasty by Dotter et al. in (3) and used in the treatment of patients with atherosclerosis. In the 1970s, it was found that percutaneous intracoronary balloon angioplasty could be used as a non-surgical dilatation method to improve coronary artery stenosis. In the 1980s, BMS was developed and gradually used to treat coronary artery disease and peripheral artery disease. Until the early 21st century, the combination of drug and stent treatment technology matured and the therapeutic safety of DES was effectively verified, to the point that the effectiveness of DEB for in-stent restenosis has been confirmed and gradually expanded to the treatment of more arterial system diseases.

In 1964, Dotter et al. proposed percutaneous transluminal angioplasty to treat inoperable arteriosclerosis stenosis (3). In 1978, Grüntzig et al. developed the expandable balloon technique to propose intraluminal dilation of coronary artery stenosis, which was successfully implemented in clinical practice. Although balloon dilation was limited at that time due to the potential for restenosis with long-term use, it cannot be denied that balloon dilation showed better short-term efficacy (4–6). In the 1980s, Sigwart et al. introduced bare metal stents (BMS) for the treatment of coronary and peripheral artery stenosis, significantly reducing restenosis and occlusion after angioplasty (7). However, the presence of BMS in the vessel lumen for an extended period may lead to stent thrombosis and worsen the condition. It wasn't until the early 21st century that drug-eluting stents (DES) were developed, greatly improving the prevention of stent thrombosis. Rensin et al.'s study, involving 15 Brazilian patients with an average age of 60, demonstrated minimal adverse events following the use of sirolimus drug-eluting stent (8, 9), although in-stent restenosis still remained a concern. In 2003, Scheller et al. conducted an animal model study mixing Paclitaxel and iopromide, revealing effective inhibition of in-stent restenosis (10). They suggested the use of paclitaxel drug balloons as a novel approach for preventing and treating restenosis the following year (11). The 2014 ESC/EACTS guidelines for myocardial revascularization have recommended considering drug-eluting balloons for restenosis (12). Now, the safety and efficacy of drug-eluting balloons have been confirmed and are gradually being utilized in clinical treatment. In recent years, with advancements in technology and research emphasis, drug balloons are no longer limited to restenosis treatment, but also applied to various arterial system diseases. Additionally, new material balloons such as nano drug balloons and micro-needle drug balloons have shown promising therapeutic effects and healing properties in experiments. It is expected that further balloon technologies will be implemented in clinical treatment as technology continues to advance in the future (Figure 1).

### 1.2 Drug eluting balloon production process evolution

The drug-eluting balloon is composed of three main components: the active substance, excipient, and balloon. These components are combined through specific production processes to meet clinical requirements, such as continuous drug delivery at therapeutic doses, long-term maintenance of drug concentration in blood vessel walls, and non-toxicity to the body (13). Various methods exist for attaching drugs to balloons, including spraying, dipping, micropipetting, and using nanoparticles to imprint the drug on the balloon surface. Among these methods, nanoparticle technology stands out due to its ability to encapsulate the drug, firmly attach it to the balloon surface, and achieve controlled release only in the fluid environment at the target site (14, 15). Continuous innovation in coating technology has led to better drug release mechanisms, such as microcapsule coatings, hydrogel coatings, polymer-free coatings, immediate-release coatings, bioadhesive coatings, and multilayer coatings. These advancements enable more uniform and stable wrapping of drugs on the balloon surface, reduce drug loss during delivery, and improve drug delivery to the target site (16, 17). When selecting a drug, it's essential to consider its lipophilicity since the balloon must deliver a large dose of the drug to the target vessel surface within a short time. Ideally, a lipophilic drug is preferred to ensure effective absorption by the vessel wall and tissue retention (18). Currently, paclitaxel is the most commonly used drug for Drug-Eluting Balloons (DEB), but alternative options are being investigated due to potential risks. A comparative trial between paclitaxel-coated balloons and sirolimus-coated balloons showed no significant difference in short-term clinical outcomes, indicating that DEB utilizing Moss family drugs could potentially replace paclitaxel-coated balloons (19). The choice of excipients is equally important as the antiproliferative drugs. During balloon dilation, excipients allow the drug to attach completely to the endothelium and mucus layer. They also ensure stability before gradual controlled release, enabling long-term treatment (20). Commonly used excipients in clinical practice include urea, iopromide, tributyl acetyl citrate, and polyester-based polymers (21). Urea facilitates penetration of the lipophilic portion or drug into the arterial wall, iopromide acts as a hydrophilic spacer, and lipophilic lubricant-type excipients reduce friction between the balloon, polymer layer, and vessel. However, it's important to consider potential vascular inflammation and allergic reactions associated with excipients. In this regard, a research team has developed an excipient-free paclitaxel nano-needle crystal drug balloon, which improves biocompatibility and reduces the risk of distal microvascular embolism by using a unique excipient-free design and a breakthrough of the drug crystal diameter to the nanometer level. In comparison with the current clinical evidence of Symplex drug balloon, it was found that there was no difference in safety and efficacy between the two, which fully proved that the excipient-free paclitaxel nano-needle crystal drug balloon can improve the safety of use and still take into account the excellent efficacy, providing a new development direction for the production of drug balloons. Balloons as material carriers have high requirements for flexibility, mechanical strength, and thickness, the original balloons were made of polyvinyl chloride, but today, balloons are mainly made of thermoplastic polymers, there are also balloons made of cross-linked polyethylene, polypropylene, polyamide, and polyester, depending on the clinical purpose, the balloons can be made in different lengths and diameters (16). Various improvements have been made to the balloon itself to address the problems of short inflation times, inefficient drug delivery, and loss of drug flow. Linear micropatterned drug-eluting balloons improve the efficiency and accuracy of intravascular drug delivery to the target lesion through higher contact area and more effective “drug ramming”, so as to create a fuller contact between the balloon and the luminal side of the vascular tissue (22). Microneedle Drug Eluting Balloon, which combines a 34G micro-needle with a balloon catheter based on a specific array to better distribute the drug in the vessel wall by direct injection, but due to balloon volume limitations, the needle needs to be further improved and reduced (23). At the same time, it has been proposed that an external protective sleeve can be used on the drug balloon to prevent drug loss during administration, and it has been demonstrated by in-vitro simulated dosing tests and animal tests that this method can reduce the loading of paclitaxel on the drug balloon to 30% of the mainstream loading and transfer a large amount of paclitaxel to the vessel wall, and it can be detected throughout the 90-day animal experimental study, this technique will be expected to reduce the possibility of systemic drug toxicity due to the use of paclitaxel drug balloon (24). In recent years, due to the advantages of drug balloons in clinical practice, more and more research has been devoted to the improvement of drug balloons, which improves the accuracy of drug release and reduces the toxicity of drugs to humans.

### 1.3 Mechanism of action of drug eluting balloon

**Figure 2:**
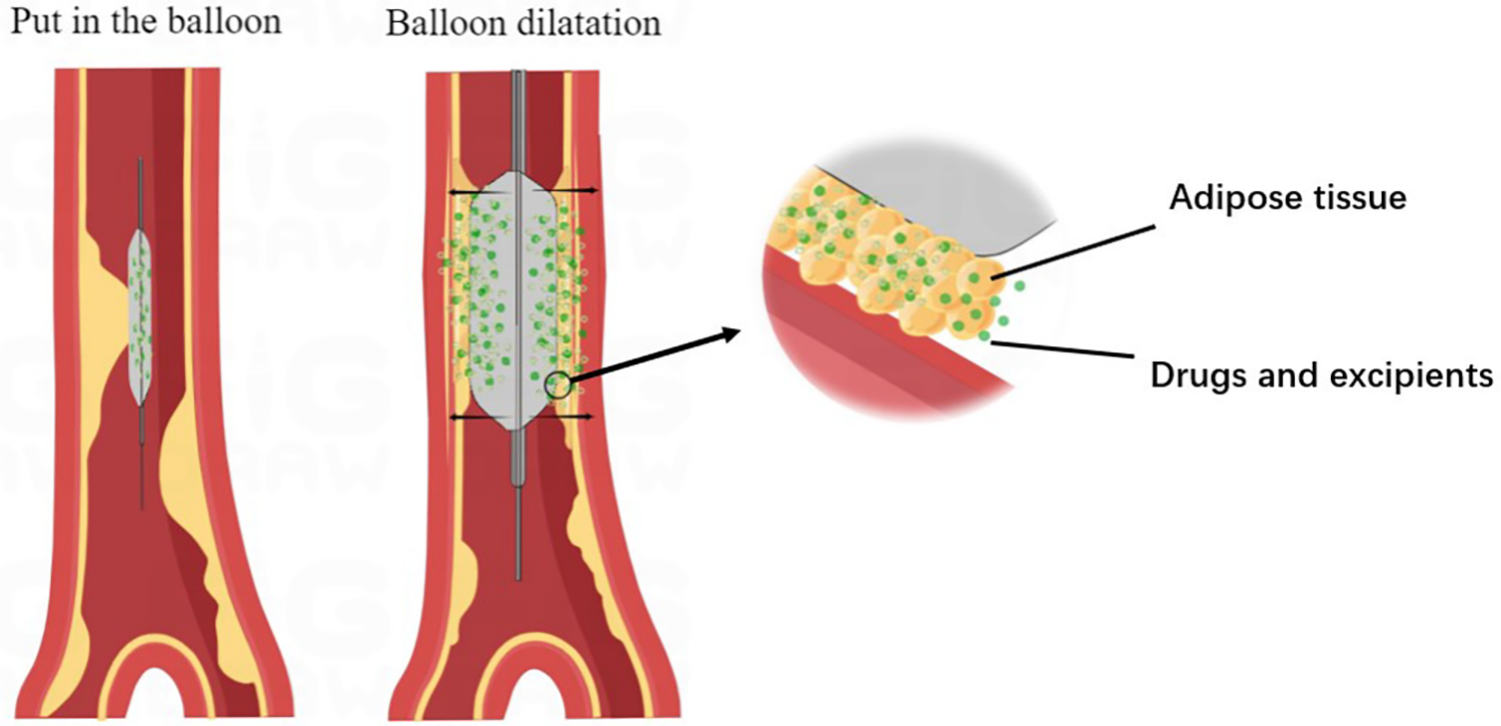
Mechanism of drug balloon action. The diagram illustrates the process of drug balloon after entering the vessel, the balloon expands at the lesion, enlarging the lumen of the vessel, and the drug inside the balloon reaches the vessel wall with the excipient through the atherosclerotic material present in the vessel wall and is absorbed by the vessel wall to act.

Before using drug-eluting balloons (DEB), it is necessary to perform pre-dilatation with various types of balloons such as normal balloon, high pressure balloon, cutting balloon, integrating balloon, or sphenoid balloon. This pre-dilatation helps reduce the occurrence of intimal dissection. The ratio of balloon diameter to vessel diameter is generally recommended to be within the range of 0.8:1–1.0:1 (21). Once adequate pre-dilatation is achieved, DEB balloons are employed to dilate the stenosed vessel. During this process, the drug is released from the balloon, allowing it to reach the lesion and penetrate into the vessel wall. This drug release exerts an inhibitory effect on intimal hyperplasia (Figure 2). Some commonly used drug-eluting balloons include DIOR DEB, PACCOCATH DEB, SeQuent DEB, and IN.PACT DEB. These balloons typically utilize Paclitaxel as the drug (25). DIOR DEB has multiple micropores on the balloon surface through which Paclitaxel can be rapidly and efficiently released. PACCOCATH DEB and SeQuent DEB both have Paclitaxel embedded in their coatings, enhancing the solubilization and transfer of the drug. However, the coating substance used in SeQuent DEB rapidly dissolves after balloon expansion. On the other hand, IN.PACT DEB is a relatively new type of balloon that contains the hydrophilic excipient, allantoin, in its coating. This excipient facilitates the release and transfer of paclitaxel to the target lesion (26–30). While Paclitaxel is commonly used as a cell proliferation inhibiting drug in drug-eluting balloons, there are other drugs being explored for their potential in this application. Cells respond differently to different concentrations of Paclitaxel, but all concentrations inhibit mitosis by targeting spindle microtubule dynamics, thereby suppressing cell proliferation. Low concentrations of Paclitaxel delay mitosis but may lead to cell death or aneuploidy. On the other hand, high concentrations of Paclitaxel block mitosis by maintaining the formation of microtubule protein complexes (31, 32). Due to the narrow therapeutic window and challenges in controlling the safe dosage of Paclitaxel, as well as suboptimal inhibition of cell proliferation, there is a growing interest in developing new drugs for drug-eluting balloons. One such drug is rapamycin, which inhibits cell proliferation by targeting the mammalian rapamycin target protein signaling pathway (33). Studies have shown that rapamycin exhibits better inhibition of coronary proliferation compared to Paclitaxel (34), although it may have limited effectiveness in certain lesions. Apart from Paclitaxel and rapamycin, other drugs like sirolimus and everolimus are also being investigated for their ability to stabilize blood vessels and enhance the effect of drug-eluting balloons (35).

## 2 The application of drug eluting balloon in arterial circulatory diseases

**Figure 3:**
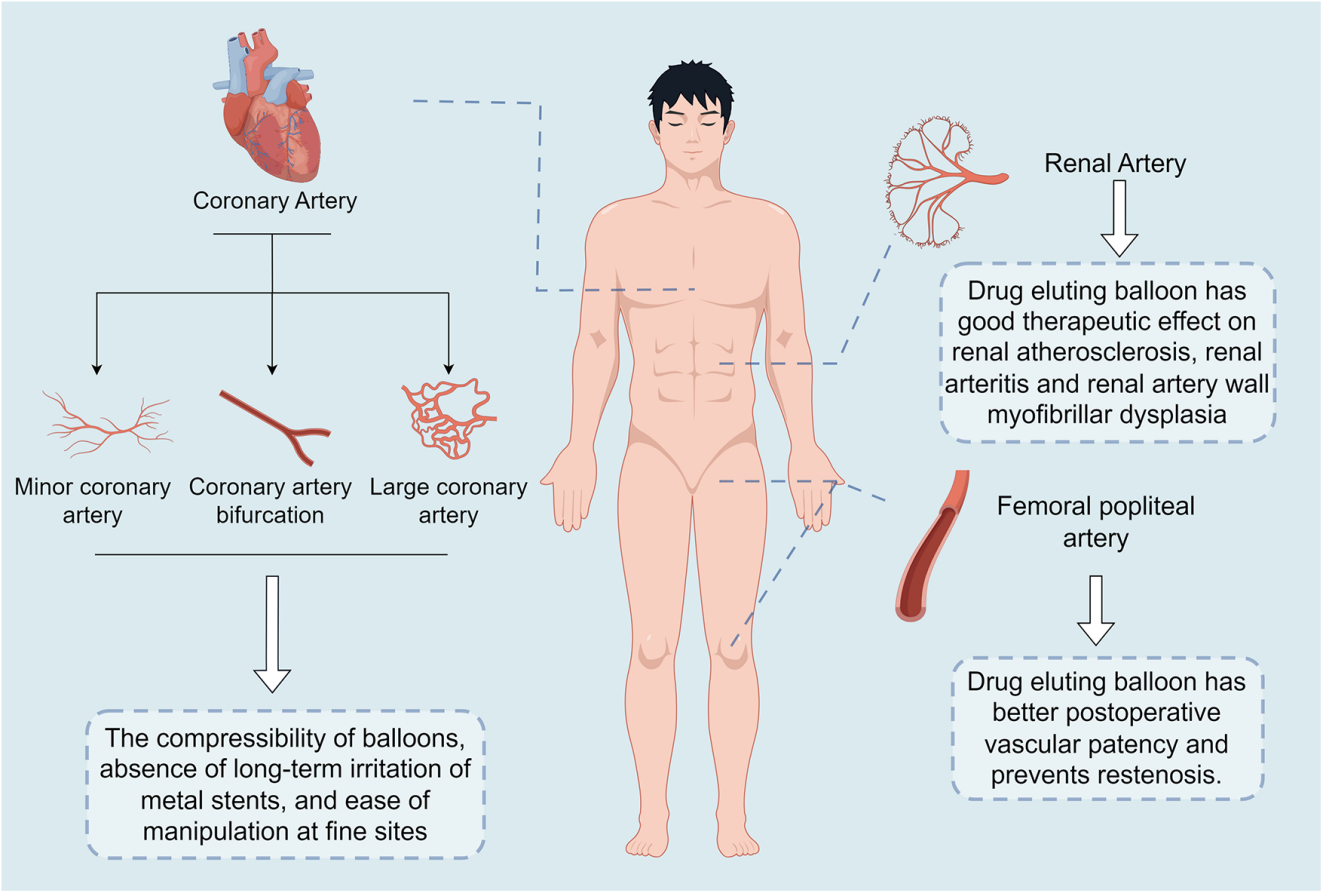
Key findings of the drug-eluting balloon. The main points outlined in this graphic are: in coronary artery disease, the drug-eluting balloon is compressible, has no long-term irritation from metal stents, and is easy to manipulate in delicate areas; in renal artery disease, the drug-eluting balloon has been shown to have good therapeutic efficacy in renal artery atherosclerosis, renal arteritis, and renal arterial wall myofibrillar dysplasia; and in femoropopliteal artery disease, the drug-eluting balloon has resulted in improved postoperative patency and prevention of restenosis.

Until now, interventional procedures have been the most common treatment option for cardiovascular disease, DEB is one of the latest technologies developed as a new clinical treatment modality for coronary artery lesions and peripheral artery disease. Compared to traditional balloon and trans-stenting techniques, DEB can be used in combination with BMS or alone, offering advantages such as uniform drug delivery to the vessel wall, no metallic foreign body residue, reduced antiplatelet treatment time, and reduced restenosis rates (11, 36–38). At present, there are numerous studies showing that DEB has better results in the treatment of coronary artery disease and peripheral vascular disease. Especially in the treatment of coronary artery disease, DEB has better long-term treatment effect than BMS and DES techniques (39) (Table 1 and Figure 3).

**Table 1:** Application examples of drug eluting balloon in arterial circulatory diseases.

Diseases		Researchers	Outcomes
Coronary artery disease	Minor coronary artery disease	Latib et al. (40), Jeger et al. (41)	Patients on DEB had significantly lower rates of restenosis and adverse cardiac events than those on paclitaxel DES, and DEB treatment was not less effective than DES treatment
		Cortese et al. (42)	Patients in the DEB group had significantly lower late lumen loss and a significantly lower incidence of myocardial infarction and thrombosis than in the EES group
		Unverdorben et al. (43)	Patients in the DEB treatment group had a significantly lower incidence of adverse events than after DEB + BMS treatment
	Coronary artery bifurcation lesions	Mathey et al. (44), Kleber et al. (45), Liu et al. (46), Jing et al. (47), Kitani et al. (48)	DEB causes minimal branch damage, and is significantly superior to conventional balloon angioplasty and DES therapy in the treatment of coronary bifurcation lesions
	Large coronary artery diseases	Yu et al. (49), Rosenberg et al. (50), Lu et al. (51), Wei et al. (52), Hu et al. (53)	The rate of late adverse cardiovascular events was lower in the large-vessel lesion group, and the rate of long-term adverse cardiovascular events treated by DEB was better than DES
	Chronic total occlusion of coronary arteries	Köln et al. (54)	Better outcomes with DEB treatment
	Myocardial infarction	Vos et al. (55), Scheller et al. (56)	The efficacy of DEB for ST-segment and non-ST-segment elevation myocardial infarction was similar to the rest of the treatment modalities
Peripheral artery elution	Femoral popliteal artery disease	Kayssi et al. (57), Tepe et al. (58), Liistro et al. (59), Bausback et al. (60)	DEB is more effective in high-risk femoropopliteal artery injuries and can improve lower extremity arterial patency and binary restenosis rates
	Renal artery disease	Takahashi et al. (61), Yamamoto et al. (62), Bi et al. (63), Li et al. (64), Kozlova et al. (65)	DEB is a safe and effective treatment for disease caused by renal artery stenosis, TRAS
Intra-stent restenosis lesions		Virga et al. (66), Samady et al. (67), Alfonso et al. (68), Giacoppo et al. (69)	DEB treatments show good therapeutic results and low recurrence rates

DEB, drug eluting balloon; DES, drug eluting stent; EES, everolimus eluting stent; TRAS, transplant renal artery stenosis; FMD, fibromuscular dysplasia.

### 2.1 Applications in coronary artery lesions *in situ*

#### 2.1.1 Minor coronary artery disease

Percutaneous coronary intervention (PCI) has emerged as the primary treatment modality for coronary artery disease (CAD) (70). However, the management of minor coronary artery lesions with a small vessel diameter (<2.8 mm) poses challenges due to technical limitations, resulting in high restenosis rates following treatment. Currently, there is an absence of suitable stents available for clinical use in these cases, making minor coronary artery disease a significant concern in clinical practice. Advancements in balloon technology have sparked increased interest in the use of drug-coated balloons (DCBs) for treating small coronary artery disease. DCBs offer advantages such as improved compressibility, absence of long-term metal stent irritation, and ease of manipulation at fine sites. The use of balloons allows for easy access to the lesion and effective dilation of stenotic vessels. Additionally, drugs carried by the balloons can be uniformly released over a short period, resulting in favorable therapeutic outcomes. A 6-month BELLO study conducted by Latib et al. in 2012 involved 182 patients with an average age exceeding 64 years (40). The study demonstrated that paclitaxel DCBs were associated with significantly lower restenosis rates and adverse cardiac events compared to patients treated with paclitaxel-eluting stents (DES). Nevertheless, it's important to note that the study's short observation period and small sample size only provide insights into the short-term efficacy of DCB treatment for small coronary artery disease. Contrasting these findings, Jeger et al. conducted a large-scale, long-term study involving 758 participants with an average age exceeding 65 years (41). Their results indicated similar rates of mid-cardiovascular adverse events between patients treated with DCBs and those treated with DES for small native coronary artery disease. Thus, suggesting that DCB treatment is not less effective than DES treatment. Further supporting the effectiveness and safety of DCBs in the treatment of small coronary artery disease, Cortese et al. conducted a 6-month clinical trial involving 232 elderly patients in Europe (42). The study demonstrated that paclitaxel DCBs were more effective than everolimus-eluting stents (EES). This was evidenced by significantly lower late lumen loss, as well as a reduced incidence of myocardial infarction and thrombosis in the DCB group. Researchers have also evaluated the therapeutic effects of combining drug balloons with other techniques. Unverdorben et al. compared the incidence of adverse cardiovascular events in 120 elderly German patients with coronary small vessel disease after DCB therapy or DCB + BMS therapy (43). Their findings suggested that DCB therapy alone was significantly superior to combined therapy, possibly due to the fact that bare-metal stents (BMS) stimulate the vessel wall and increase target cell blood flow reconstruction, hindering lesion healing.

#### 2.1.2 Coronary artery bifurcation lesions

Coronary artery bifurcation lesions refer to the blockage of the coronary artery system at its bifurcation, which is a common form of coronary artery disease. The treatment of these lesions has been studied extensively over time. With the advancement of balloon technology, the use of balloons for treating bifurcation lesions is gaining attention. Authoritative organizations like the European Bifurcation Club and the International DEB Consensus Group have proposed the feasibility of percutaneous coronary intervention as a treatment method for coronary bifurcation disease (21, 71–73). Mathey et al. successfully treated 28 stable angina patients with coronary bifurcation lesions using paclitaxel DEB, with low incidence of adverse events. This suggests the feasibility of DEB for treating coronary bifurcation lesions. Another study conducted by Kleber FX included 128 patients over the age of 65, treated in Germany, and found the effectiveness of DEB for coronary bifurcation disease through the randomized multicenter PEPCAD-BIF trial (44, 45). However, the small sample sizes (28 and 64 patients) in both studies, including various lesion sites, limit the ability to draw definite conclusions. For left main trunk bifurcation lesions, Liu et al. observed lower incidence of adverse cardiovascular events in the DEB treatment group compared to the DES group in 85 patients. This suggests the effectiveness and safety of treating left main trunk bifurcation lesions solely with DEB (46). The importance of side branches becomes evident when the main coronary artery is blocked. Jing et al. studied DEB for treating coronary artery side branch bifurcation lesions and found that DEB was significantly more effective than conventional balloon angioplasty in 222 elderly patients (47). Therefore, drug balloons have shown effectiveness in treating both collateral and main coronary arteries with bifurcation lesions. Furthermore, combining directional coronary atherectomy with DEB angioplasty for bifurcation lesions in coronary arteries can yield positive clinical results with minimal branch damage, making it a potential stent-free percutaneous coronary intervention strategy (48). Overall, the use of drug balloons not only allows immediate opening of the bifurcation vessel but also reduces the occurrence of major adverse cardiovascular events, improving the long-term prognosis of patients (74).

#### 2.1.3 Large coronary artery disease

Large coronary vessels (≥2.8 mm) encompass the right coronary artery, left anterior descending branch, and left circumflex branch. Coronary large vessel lesions represent a common form of coronary artery disease. These lesions, located within the coronary arteries, can manifest as diffuse, obstructive, or bifurcation lesions, highlighting the extensive involvement of large vessels. Consequently, research and treatment of large coronary vessels face numerous vascular limitations. With advancements in research techniques, an increasing number of studies have demonstrated the efficacy of drug-eluting balloons (DEB) in treating coronary large vessel lesions (75, 76). In a prospective study comparing patients with large and small coronary artery lesions after DEB treatment, the rate of major adverse cardiovascular events was lower in the large-vessel lesion group, with no reported fatalities (49). By dividing 234 patients with new-onset coronary artery disease into large-vessel and small-vessel groups based on vessel size and administering DEB treatment to both groups, Rosenberg et al. observed a target lesion revascularization rate of 3.8% in small vessels and 1.0% in large vessels after 9 months. The conclusion drawn was that DEB exhibited comparable therapeutic effects in both groups, with no statistically significant difference (50). Additionally, in a study conducted by Lu et al., 92 patients with an average age of 52 underwent DEB treatment for coronary macroangiopathy, yielding a major adverse cardiovascular event rate and target lesion revascularization rate of 4.3% in the short-term. These findings confirmed the safety and efficacy of DEB monotherapy for short-term treatment of coronary macroangiopathy. However, due to the limited observation period and small sample size, the confidence level of this conclusion was low (51). To investigate the long-term clinical effects of DEB, Wei et al. performed a randomized trial involving 100 patients with large coronary artery lesions who received either DEB or drug-eluting stents (DES). The late lumen loss and long-term incidence of major adverse cardiovascular events were monitored in both groups. Although the difference in late lumen loss between the two groups was marginal, DEB exhibited slightly superior distant positive vascular remodeling compared to DES (52). In a study by Hu et al., 119 patients with large-vessel lesions in coronary arteries, aged approximately 56 years on average, were followed up for 2 years to assess the incidence of adverse cardiovascular events. The patients were categorized into two groups: large-vessel bifurcation lesions and non-bifurcation lesions. The long-term follow-up revealed a low incidence of adverse cardiovascular events in both groups, indicating favorable long-term clinical outcomes of DEB-only treatment for large-vessel lesions in coronary arteries (53). The complex nature of large coronary artery disease has limited research in this area. While drug-eluting stents remain the primary clinical treatment for large vessel lesions, evidence suggests that DEB is a safe and effective alternative for treating large coronary artery disease, demonstrating promising short-term and long-term clinical efficacy.

#### 2.1.4 Other *in situ* disease of the coronary arteries

Besides the aforementioned coronary artery diseases, drug-eluting balloons (DEB) are also utilized in the treatment of chronic total occlusive lesions, diffuse coronary artery disease, myocardial infarction, and other related conditions. Chronic total occlusion of the coronary arteries refers to the progressive narrowing of the arterial lumen, eventually leading to complete blockage. This condition is more prevalent in patients with coronary artery disease (CAD) (77), the safety of percutaneous coronary intervention has been established for chronic total occlusive coronary artery disease (78). In a study conducted in 2013, researchers exploring the efficacy of bare-metal stents (BMS) in treating chronic total occlusion observed that the combination of paclitaxel DEB treatment was more effective (79). Subsequently, Köln et al. followed up on 34 patients with an average age of 59 who underwent DEB treatment for chronic total occlusion. Through angiography, they found that DEB angioplasty, despite the absence of stent fixation, was a feasible and well-tolerated treatment option for chronic total occlusion of the coronary arteries in patients with good pre-expansion (54). According to the 2021 recommendations by the Asia-Pacific Consensus Panel for DEB treatment of coronary artery disease, DEB treatment is indicated for various cases, including chronic total occlusion of the coronary arteries (80), this establishes the feasibility of DEB in treating chronic total occlusive coronary artery disease.

Diffuse coronary artery disease was defined on coronary angiography as long coronary segments (≥20 mm) with angiographic irregularity in the absence of significant focal stenosis. It is an important determinant of the expected outcome of percutaneous coronary intervention (81). It can occur in distal coronary arteries, descending branches, side branches, and bifurcations, and its complexity results in a poorer outcome compared with focal lesions. The use of DES alone not only has long-term metal irritation and a higher risk of late thrombosis, but also the placement of long DES stents (≥60 mm) can lead to an increased incidence of adverse events, resulting in poorer outcomes in diffuse coronary artery disease, whereas the combination of DEB and DES can limit the length of the stent to a certain extent, reduce stent loading, and decrease the incidence of the aforementioned adverse events, providing a favorable treatment for diffuse coronary artery disease (82, 83).

Myocardial infarction, a serious disease associated with poor healing and commonly occurring as a complication of cardiovascular diseases, can be categorized into ST-segment elevation infarction, where vessels are mostly completely blocked, and non-ST-segment elevation infarction, where vessels are mostly incompletely blocked (55). Percutaneous coronary artery therapy is the primary treatment choice for myocardial infarction, and available studies indicate that DEB is safe and feasible in its treatment (56, 84). In a 2019 REVELATION study involving 120 middle-aged and elderly patients with acute myocardial infarction, Vos et al. compared the efficacy of DEB and drug-eluting stents (DES) in treating ST-segment elevation myocardial infarction. They found that the mean flow reserve fraction of patients in the DEB group was similar to that of the DES group, demonstrating that DEB was not inferior to DES in this regard (85). Later, Scheller et al. conducted a similar study comparing DEB, bare-metal stents (BMS), and second-generation DES in the treatment of non-ST-segment elevation myocardial infarction. The results also indicated non-inferiority for DEB, suggesting its feasibility and safety in treating both types of myocardial infarction (86). However, due to the limited number of studies on the use of DEB in myocardial infarction, further extensive research is required to establish its safety and efficacy. Additionally, drug-eluting balloons have demonstrated effectiveness in treating coronary artery disease in combination with diabetes mellitus, diffuse disease, and calcified lesions (57, 87, 88).

The minimally invasive nature of drug-eluting balloon implantation has led to its application in the treatment of cardiovascular disease in children. Children's coronary arteries are more prone to in-stent restenosis due to their smaller vessel diameters and higher percentage of lumen loss after stent implantation compared to adults. The long-term consequences of coronary stent implantation in children remain uncertain, thus opting for a “stent-less intervention” strategy seems more favorable for the long-term prognosis of pediatric patients. Wang et al. described a case involving a 16-year-old female patient who experienced a non-ST-segment elevation infarction caused by aortitis, and subsequently underwent drug-coated balloon angioplasty for in-stent restenosis that occurred one year after coronary stent implantation (58). Xu et al. reported the successful treatment of severe stenosis of the proximal left anterior descending coronary artery caused by Kawasaki disease in a 6-year-old boy using drug-coated balloon revascularization, demonstrating the feasibility of this treatment approach in children with Kawasaki disease (59). However, further investigation is needed to determine the safety and efficacy of drug-coated balloon treatment in this particular population. Hirose et al. initially described a male patient who underwent heart transplantation for restrictive cardiomyopathy at the age of 2 years and developed severe stenosis of the proximal left anterior descending coronary artery at the age of 11 years (60). Due to the rejection reaction, the transplanted cardiac population was more susceptible to restenosis compared to the general population. Nevertheless, no restenosis was observed during the 7-month follow-up period after undergoing angioplasty with drug-coated balloons, indicating the feasibility of using them in pediatric patients undergoing heart transplantation. However, as children represent a specialized group still in an important developmental phase, their vascular alignment, size, and tolerance to antiproliferative drugs differ significantly from those of adults. The long-term efficacy and safety of drug-coated balloons in pediatric cardiovascular disease have not yet been reported, thus further research is necessary to explore the long-term outcomes.

### 2.2 Applications in peripheral arterial system lesions

Peripheral artery disease (PAD) is a condition caused by atherosclerosis in non-cardiac blood vessels, with the femoral popliteal artery being the most commonly affected (89). The 2011 ESC treatment guidelines emphasize the importance of endovascular approaches, such as percutaneous transluminal angioplasty (PTA), for PAD management (90). However, due to the high restenosis rate associated with conventional treatments, scholars are increasingly focusing on the efficacy of drug-eluting balloons (DEBs) in peripheral arterial disease. In a large-scale study conducted by Kayssi et al. in 2016, DEB treatment was found to have a superior effect on lower extremity arterial patency and binary restenosis rates compared to conventional treatments (91). Nevertheless, it is worth noting that for more severe cases such as amputation, there is no significant evidence indicating a better treatment effect of DEBs. Comparative studies have shown that DEBs exhibit advantages over conventional angioplasty and drug-eluting stents (DES). For instance, a randomized trial by Tepe et al. demonstrated significantly higher patency rates in elderly patients with popliteal arteries treated with DEBs compared to those treated with conventional PTA (92). Similarly, the DRASTICO study conducted by Liistro et al. in 2019 revealed that both DEBs and DES were effective in treating high-risk femoropopliteal artery injuries, but DEBs did not show a significant advantage over DES (61). Consistent findings were reported by Bausback et al. in a clinical study involving 150 patients (62). These findings collectively suggest that DEBs offer improved treatment outcomes in femoropopliteal artery disease, enhancing patency and reducing restenosis risks.

The renal artery, as a crucial peripheral artery, commonly experiences stenosis in renal artery disease. Atherosclerotic renal artery stenosis is the most prevalent cause, followed by aortitis and myofibrillar dysplasia of the arterial wall (63). Stenting the renal artery can cause mechanical damage, leading to intimal disruption and subsequent smooth muscle cell and intimal proliferation, resulting in in-stent restenosis (ISR). Research has indicated that ISR exhibits more rapid smooth muscle cell proliferation than original plaque cells (64). In a study by Takahashi et al., which followed 1,052 patients treated with percutaneous transluminal renal artery angioplasty for renal artery stenosis, secondary stenting was associated with a significantly higher probability of restenosis compared to balloon dilation alone (65). Consequently, finding treatments that delay ISR onset while preserving the luminal diameter of the renal artery is an important task for scholars. Drug balloons have shown potential in reducing antiplatelet drug use and mitigating the inflammatory response in patients. Patients with renal stenosis associated with aortitis experience a high restenosis rate of up to 78% after renal angioplasty; however, drug balloons offer significant delays in restenosis (93). Notably, Yamamoto et al. reported a case of a patient with aortitis-induced renal artery stenosis who underwent multiple renal angioplasty treatments before achieving blood flow velocities similar to those after two years of drug balloon therapy (94). Similar outcomes were observed in five patients with aortitis who received pharmacological balloon treatment, as reported in another study (66). Nevertheless, further research is required to fully understand the efficacy of drug balloon therapy for aortitis-related renal artery stenosis. Restenosis rates remain high in stenting for renal artery stenosis after renal transplantation (TRAS), with reported incidences of 15% for bare metal stents and 15.7% for drug-eluting stents (67). The primary mechanism underlying TRAS-related restenosis is intimal hyperplasia, and drug balloons possess inherent advantages in directly targeting this condition. A retrospective study on 14 patients with TRAS treated with drug balloons indicated that the therapy was safe and effective in the short term, as there were no significant changes in creatinine and blood pressure indexes during a 6-month follow-up period (68). However, considering the limited number of patients included in this study and the lack of a control group receiving regular balloon treatments, further validation is needed to assess its long-term effects. Fibromuscular dysplasia (FMD), characterized by abnormal cell proliferation and structural variations in the arterial wall, is the second most common cause of renal artery stenosis (69). Due to insufficient available data, the therapeutic effect of pharmacological balloon therapy for FMD requires further exploration.

### 2.3 Application in in-stent restenosis lesions

In-stent restenosis refers to the reoccurrence of stenosis following the insertion of a stent in a narrowed blood vessel for dilation. The underlying mechanism primarily involves the long-term presence of the metallic stent as a foreign body within the arterial vasculature. This presence can induce changes in vascular wall stress and inflammation, leading to fibrin deposition, migration of fibroblasts, and neointimal hyperplasia, ultimately resulting in in-stent restenosis (95). Clinical experience indicates that reusing stents increases the likelihood of in-stent restenosis. However, drug-eluting balloons (DEB) offer an alternative approach. DEBs release anti-proliferative drugs during balloon dilatation without the need for permanent stent implantation. This allows for rapid drug delivery to the diseased vessel, promoting drug absorption and effectively preventing restenosis while protecting the intima. Compared to balloon-only or stent implantation strategies, DEB significantly reduces positive vascular remodeling and subsequent inflammatory responses. Consequently, DEB has been recommended as an effective treatment for in-stent restenosis in the 2014 ESC guidelines (12). There are two common clinical types of in-stent restenosis: bare-metal stent (BMS) in-stent restenosis and drug-eluting stent (DES) in-stent restenosis. Research on DEB for in-stent restenosis continues. A prospective study by a French scholar involving 206 elderly patients with DES in-stent restenosis found a low incidence of adverse cardiovascular events in patients treated with paclitaxel DEB (96). Virga et al. observed 39 patients with in-stent restenosis of the superficial femoral artery treated with DEB for 2 years, and they reported a high patency rate with only one death during the observation period (97). Similarly, Samady et al. found favorable long-term outcomes for DEB treatment of in-stent restenosis (98). Alfonso et al. conducted a randomized study on 189 elderly patients with BMS in-stent restenosis, comparing DEB treatment group and EES treatment group. The study revealed excellent treatment results in both groups, with a significantly lower recurrence rate (99). In the DAEDALUS study by Giacoppo et al. in 2020, which involved 710 elderly patients with BMS in-stent restenosis and 1,248 elderly patients with DES in-stent restenosis treated with DEB, the researchers found that DEB was effective in treating both types of in-stent restenosis mentioned above, but BMS in-stent restenosis had a better outcome than DES in-stent restenosis (100). Both DES in-stent restenosis and BES in-stent restenosis have a 10%–20% probability of recurrence (101), and their treatment is more challenging than primary stenosis. Recent studies have shown improved efficacy of DEB for recurrent in-stent restenosis, although differences still exist when compared to implantable fine DES (102, 103). The most effective treatment option for recurrent in-stent restenosis remains to be investigated, and the possibility of in-stent restenosis after DEB treatment cannot be ignored.

## 3 Drug eluting balloon application dilemma

### 3.1 Technical difficulties with drug-eluting balloons

Although numerous studies have demonstrated the therapeutic efficacy of drug-eluting balloons (DEB), and some countries and regions have included DEB as a medical device in their medical insurance coverage (Table 2), DEB still encounters several technical challenges. Firstly, the main therapeutic effect of DEB, which involves the use of anti-proliferative drugs such as Paclitaxel, is hindered by its cytotoxicity. Different drugs exhibit varying pharmacodynamic properties, and the efficiency of drug absorption by the vascular wall is negatively correlated with the lipid content of the wall. Consequently, diseases like atherosclerosis that increase the lipid content of the vascular wall also affect drug absorption efficiency. To counter this, DEB often employs drugs with higher lipophilicity, with Paclitaxel being the most commonly used anti-proliferative drug at present (104). But one study discovered that paclitaxel transportation is significantly impeded in the presence of thrombosis in blood vessels (105). Moreover, strict dose control is required for the use of Paclitaxel, generally within the range of 3–5 µg/mm^2^ (106). Lower doses decrease the antiproliferative effect on the vessel wall, while higher doses may cause cytotoxicity after prolonged exposure (107–109). Furthermore, Paclitaxel exhibits poor efficacy, a narrow therapeutic window, and low safety among various anti-proliferative drugs. Consequently, some studies have proposed the replacement of Paclitaxel with rapamycin, which offers similar anti-proliferative effects and a safer drug dosage. However, the inhibitory effect of rapamycin and its derivatives on intravascular lesions is still under investigation (18, 104). Additionally, Wessely et al. found that when comparing the performance of Paclitaxel and rapamycin on drug-eluting stents (DES), rapamycin resulted in a higher restenosis rate and significantly greater late lumen loss (110). Similarly, Alfonso et al. compared the efficacy of Paclitaxel and everolimus in treating patients with in-stent restenosis and found that everolimus was significantly more effective than Paclitaxel (111). Although Paclitaxel carries a risk of cytotoxicity, the drug balloon used in clinical practice is a finished product that does not require physicians to control the drug dosage. Consequently, the clinical manifestation of Paclitaxel cytotoxicity is reduced, thus promoting its use in clinics, despite it not being the optimal choice. Secondly, the selection of excipients is crucial. Currently, anti-proliferative drugs in DEB are typically used in combination with excipients to minimize drug loss during balloon transport. Common excipients include hydrophilic substances such as urea and iopromide. While highly hydrophilic substances effectively prevent the loss of highly lipophilic drugs during transport, some studies have indicated that urea and iopromide tend to shed their coating when transported within the vasculature for extended periods. This shedding increases the rate of drug loss and diminishes efficacy (35, 112, 113). Lastly, apart from optimizing Paclitaxel drugs and excipients, precise control of balloon dilation pressure is also essential. Animal experiments conducted by Stolzenburg et al. demonstrated that higher inflation pressure promotes the transfer of Paclitaxel in atherosclerosis (114). However, excessive inflation pressure can lead to plaque rupture, aggravate vascular injury, increase the risk of vascular dissection, and cause in-stent restenosis. Conversely, low inflation pressure results in insufficient contact between the balloon and the vessel wall, reducing drug efficacy. While prolonging the contact time between the balloon and the vessel wall can enhance drug absorption, it also increases the risk of vascular injury (115, 116). Consequently, resolving the issues associated with balloon expansion pressure and achieving optimal contact time between the balloon and the vessel wall has become a recent focus of research. Numerous micron and nano new materials may offer potential solutions to this problem.

**Table 2:** Approval of vascular DEB medical devices in some countries.

Name	Production company	Application area	Approving country (institution)	Included in approved country medical insurance?
Drug balloon dilatation catheter	Shanghai MicroPort Endovascular MedTech (Group) Co., Ltd.	Femoral artery, popliteal artery	China	Yes
Coronary drug balloon dilatation catheter	DK Medical Technology Co., Ltd.	Coronary artery	China	Yes
PTA drug balloon dilatation catheter	Lutonix, Inc.	Unknown	China	Yes
SurVeil Drug-Coated Balloon	Surmodics, Inc.	Femoral artery, popliteal artery	USA(FDA)	Unknown
Stellarex 0.035″ OTW Drug-coated Angioplasty Balloon	Philips Image Guided Therapy Corporation	Femoral artery, popliteal artery	USA(FDA)	Unknown
Chocolate Touch Paclitaxel Coated PTA Balloon Catheter	TriReme Medical, LLC	Femoral artery, popliteal artery	USA(FDA)	Unknown

Information Sources: Available online at: https://mdenter.bcpmdata.com/, www.nmpa.gov.cn, www.fda.gov. USA: The United States. FDA, Food and Drug Administration.

### 3.2 Drug eluting balloon complication management

DEB, a novel balloon technology, offers significant advantages in the treatment of arterial circulatory disorders. However, it's important to acknowledge that complications such as microthrombosis and vascular dissection can occur during its utilization. Despite being infrequent, these complications cannot be disregarded considering their serious consequences. The combination of lipophilic antiproliferative drugs with hydrophilic excipients holds the potential for producing a highly crystalline coating that is unstable and prone to particle formation during crystallization. Particularly, the combination of highly hydrophilic excipients like urea and iopromide with lipophilic drugs such as paclitaxel can lead to easier dissolution of the coating during balloon transport, resulting in the formation of numerous particles. This occurrence is especially prevalent during balloon expansion, which increases the risk of emboli formation when these particles reach downstream vessels (112, 117). Kelsch et al. observed in an animal model that at least 25%–35% of the paclitaxel drug was shed and thrombotic occlusion was observed during the entry of DEB with urea and iopromide as excipients into the vasculature to the site of lesion initiation (118). To address this issue, Gongora CA proposed the use of a more hydrophobic excipient like BTHC to enhance the integrity of the balloon coating, subsequently reducing particulate production. In their investigation, they compared three different DEB technologies—paclitaxel urea DEB, paclitaxel polysorbate DEB, and paclitaxel BTHC DEB—in a porcine model. The results demonstrated that paclitaxel BTHC DEB produced nearly ten times fewer particles compared to paclitaxel urea DEB and paclitaxel polysorbate DEB (119). In addition to employing more hydrophobic excipients for minimizing particulate production, alternative methods have been proposed. One such method involves placing the drug and excipient within a folded balloon, reducing the exposed area of the drug coating during transport. Once the balloon reaches the lesion site, it expands fully to ensure complete drug exposure (11). These two improved methods are currently under investigation. Whether a more hydrophobic excipient or a folded balloon approach is chosen, it is evident that a high level of technological proficiency is required. However, questions regarding the potential delayed release of the drug upon contact with the vessel wall and the ability to accurately release the drug still necessitate further confirmation through additional studies.

Since pre-dilatation of the vessel is necessary before using DEB, it is essential to consider the impact of DEB being stent-free. In cases where pre-dilatation is insufficient, the vessel may lose stenting support and experience vascular elastic retraction. Conversely, excessive pre-dilatation can lead to vessel dissection, resulting in restricted blood flow (120, 121). Balloon angioplasty functions by stretching atherosclerotic arteries, which often causes vascular injury leading to dissection (122). The anatomical structure of coronary arteries typically comprises intima, media, and adventitia. Arterial dissection occurs when layers of the arterial wall separate, forming a false lumen between the intima and media or between the media and adventitia (123). Separation of the epicardium is accompanied by the formation of a false lumen, and this stripping reduces or obstructs blood flow, which reduces the rate of recanalization of the target diseased vessel at the site of the lesion, making healing very difficult, and if the entrapment remains untreated for a long period of time, it can lead to endothelial hyperplasia and restenosis due to prolongation of the inflammatory process (124, 125). In contrast, in a THUNDER study it was found that treatment with DEB PCI did not require implantation of a stent as long as the entrapment does not lead to acute blood flow restriction, stent implantation is not required (126). According to the Delphi Expert Consensus, stenting is recommended for post-balloon angioplasty dissection in cases involving reduced lesion vessel diameter, impaired blood flow, or poor morphology (127). The Tack Endovascular System (Intact Vascular, Wayne, Pennsylvania) is a novel device that has been utilized by Gray WA to treat 213 patients and assess dissection repair outcomes. The study confirmed the safety and efficacy of the Tack Endovascular System in focal dissection repair (128). Furthermore, Kobayashi N observed 319 elderly patients with vascular dissection in the femoropopliteal artery and found higher restenosis rates in patients with severe dissection. This finding suggests that the generation of vascular dissection may be a significant factor contributing to in-stent restenosis occurrence (129). Currently, BMS in-stent restenosis and DES in-stent restenosis are common occurrences, with neointimal hyperplasia and vascular retraction being mechanisms associated with in-stent restenosis. This is especially prominent in patients with diabetes and inflammation (130). Although DEB use has shown a reduction in the incidence of in-stent restenosis, it is not entirely avoided. Some studies have unveiled that DEB is less effective than repositioning DES for treating restenosis caused by DES (100, 131). However, this challenge can potentially be addressed through the combination of DEB with biodegradable stents. Such a combination eliminates the need for permanent stent placement, and the use of biodegradable stents can prevent short-term vessel elastic retraction effectively, consequently reducing late in-stent restenosis (75). Currently, DEB combined with DES is the predominant approach in clinical practice, while BMS is rarely used due to its strong irritation of the vessel wall. There is limited research on the combined use of both techniques, and the safety and efficacy of such combinations require further investigation.

Observation of disease after DEB treatment can be used as a means to assess disease healing. Invasive and non-invasive methods are usually available, and the commonly used invasive methods include coronary angiography and optical coherence tomography. Coronary angiography can be used to compare the changes in vessel patency and stenosis before and after treatment, and can be used for localized treatment (132, 133). Optical coherence tomography is a new type of catheter-based invasive imaging modality, which uses infrared light rather than ultrasound, and can clearly reflect arterial plaques and blood clots (134). Non-invasive methods include intravascular ultrasound, coronary CT, and cardiac magnetic resonance. Intravascular ultrasound used to be the gold standard for evaluating stent placement and stent-vessel response, and it can use ultrasound to determine the structure of the vascular lumen as well as blood flow, but it has the disadvantages of more stenting artifacts and inability to distinguish small neoplastic endothelial tissues (135, 136). Overall, both types of assessment methods have their own advantages, and clinical use should be based on the patient's condition to choose the appropriate assessment method.

## 4 Conclusion

Drug-eluting balloons, as a new interventional technique, can avoid the hazards associated with stent implantation. There have been numerous clinical trials demonstrating the safety and efficacy of drug-eluting balloons in the treatment of coronary artery disease and lower extremity artery disease, and they are expected to be used in larger vessel and peripheral vascular diseases. There are also a large number of trials comparing drug-eluting balloons with other interventional techniques that demonstrate the advantages of drug-eluting balloons in maintaining vessel patency and reducing the rate of in-stent restenosis. However, the current studies on drug-eluting balloons are characterized by small sample size and short observation time, and the technical shortcomings in the clinical use of drug-eluting balloons may lead to adverse conditions such as particulate matter and vessel dissection, therefore, more studies on the safety of drug-eluting balloons are needed. By reviewing the progress of drug-eluting balloon research mentioned above, we can provide new ideas for the treatment of more arterial system diseases.

## References

[1] Yuan Y Li SM Zhu FS Zhi XL Ji XM. Correlative study of carotid transient ischemic attacks and intracranial or extracranial angiostenosis. Zhong Nan Da Xue Xue Bao Yi Xue Ban. (2008) 33:751–4. 18772519

[2] Xu X Na N Pan X Wang K Ma A Wang Y Association of TLR4 gene polymorphisms with large artery atherosclerotic stroke and vascular bed selectivity of atherosclerotic lesions. Zhonghua Yi Xue Yi Chuan Xue Za Zhi. (2014) 31:455–61. 10.3760/cma.j.issn.1003-9406.2014.04.009 25119909

[3] Dotter CT Judkins MP. Transluminal treatment of arteriosclerotic obstruction. Description of a new technic and a preliminary report of its application. Circulation. (1964) 30:654–70. 10.1161/01.cir.30.5.654 14226164

[4] Gruntzig AR Senning A Siegenthaler WE. Nonoperative dilatation of coronary-artery stenosis: percutaneous transluminal coronary angioplasty. N Engl J Med. (1979) 301:61–8. 10.1056/NEJM197907123010201 449946

[5] Louis EL Provan JL Gray RR Grosman H Ameli FM Elliott DS. Percutaneous transluminal angioplasty in peripheral vascular disease: a review. Can Fam Physician. (1982) 28:291–4. 21286052 PMC2306349

[6] Gruntzig A. Transluminal dilatation of coronary-artery stenosis. Lancet. (1978) 1:263. 10.1016/s0140-6736(78)90500-7 74678

[7] Sigwart U Puel J Mirkovitch V Joffre F Kappenberger L. Intravascular stents to prevent occlusion and restenosis after transluminal angioplasty. N Engl J Med. (1987) 316:701–6. 10.1056/NEJM198703193161201 2950322

[8] Liistro F Colombo A. Late acute thrombosis after paclitaxel eluting stent implantation. Heart. (2001) 86:262–4. 10.1136/heart.86.3.262 11514475 PMC1729898

[9] Rensing BJ Vos J Smits PC Foley DP van den Brand MJ van der Giessen WJ Coronary restenosis elimination with a sirolimus eluting stent: first European human experience with 6-month angiographic and intravascular ultrasonic follow-up. Eur Heart J. (2001) 22:2125–30. 10.1053/euhj.2001.2892 11686669

[10] Scheller B Speck U Schmitt A Bohm M Nickenig G. Addition of paclitaxel to contrast media prevents restenosis after coronary stent implantation. J Am Coll Cardiol. (2003) 42:1415–20. 10.1016/s0735-1097(03)01056-8 14563585

[11] Scheller B Speck U Abramjuk C Bernhardt U Bohm M Nickenig G. Paclitaxel balloon coating, a novel method for prevention and therapy of restenosis. Circulation. (2004) 110:810–4. 10.1161/01.CIR.0000138929.71660.E0 15302790

[12] Authors/Task Force members, Windecker S Kolh P Alfonso F Collet JP Cremer J Falk V 2014 ESC/EACTS guidelines on myocardial revascularization: the task force on myocardial revascularization of the European society of cardiology (ESC) and the European association for cardio-thoracic surgery (EACTS)Developed with the special contribution of the European association of percutaneous cardiovascular interventions (EAPCI). Eur Heart J. (2014) 35:2541–619. 10.1093/eurheartj/ehu278 25173339

[13] Gertz ZM Wilensky RL. Local drug delivery for treatment of coronary and peripheral artery disease. Cardiovasc Ther. (2011) 29:e54–66. 10.1111/j.1755-5922.2010.00187.x 20553281

[14] Petersen S Kaule S Stein F Minrath I Schmitz KP Kragl U Novel paclitaxel-coated angioplasty balloon catheter based on cetylpyridinium salicylate: preparation, characterization and simulated use in an in vitro vessel model. Mater Sci Eng C Mater Biol Appl. (2013) 33:4244–50. 10.1016/j.msec.2013.06.021 23910339

[15] Dugas TR Brewer G Longwell M Fradella T Braun J Astete CE Nanoentrapped polyphenol coating for sustained drug release from a balloon catheter. J Biomed Mater Res B Appl Biomater. (2019) 107:646–51. 10.1002/jbm.b.34157 30091513

[16] Rykowska I Nowak I Nowak R. Drug-eluting stents and balloons-materials, structure designs, and coating techniques: a review. Molecules. (2020) 25:4624. 10.3390/molecules25204624 33050663 PMC7594099

[17] Ang H Koppara TR Cassese S Ng J Joner M Foin N. Drug-coated balloons: technical and clinical progress. Vasc Med. (2020) 25:577–87. 10.1177/1358863X20927791 32634046

[18] Xiong GM Ang H Lin J Lui YS Phua JL Chan JN Materials technology in drug eluting balloons: current and future perspectives. J Control Release. (2016) 239:92–106. 10.1016/j.jconrel.2016.08.018 27554032

[19] Cortese B Caiazzo G Di Palma G De Rosa S. Comparison between sirolimus- and paclitaxel-coated balloon for revascularization of coronary arteries: the SIRPAC (SIRolimus-PAClitaxel) study. Cardiovasc Revasc Med. (2021) 28:1–6. 10.1016/j.carrev.2021.04.013 33888418 PMC8373518

[20] Zou W Cao G Xi Y Zhang N. New approach for local delivery of rapamycin by bioadhesive PLGA-carbopol nanoparticles. Drug Deliv. (2009) 16:15–23. 10.1080/10717540802481307 19555304

[21] Jeger RV Eccleshall S Wan Ahmad WA Ge J Poerner TC Shin ES Drug-coated balloons for coronary artery disease: third report of the international DCB consensus group. JACC Cardiovasc Interv. (2020) 13:1391–402. 10.1016/j.jcin.2020.02.043 32473887

[22] Lee K Lee SG Jang I Park SH Yang D Seo IH Linear micro-patterned drug eluting balloon (LMDEB) for enhanced endovascular drug delivery. Sci Rep. (2018) 8:3666. 10.1038/s41598-018-21649-7 29507314 PMC5838243

[23] Lee K Lee J Lee SG Park S Yang DS Lee JJ Microneedle drug eluting balloon for enhanced drug delivery to vascular tissue. J Control Release. (2020) 321:174–83. 10.1016/j.jconrel.2020.02.012 32035908

[24] Zhang T Guo G Yang L Wang Y. An ultralow dose paclitaxel coated drug balloon with an outer protective sheath for peripheral arterial disease treatment. J Mater Chem B. (2021) 9:2428–35. 10.1039/d0tb02720k 33624663

[25] Bukka M Rednam PJ Sinha M. Drug-eluting balloon: design, technology and clinical aspects. Biomed Mater. (2018) 13:032001. 10.1088/1748-605X/aaa0aa 29227279

[26] Posa A Nyolczas N Hemetsberger R Pavo N Petnehazy O Petrasi Z Optimization of drug-eluting balloon use for safety and efficacy: evaluation of the 2nd generation paclitaxel-eluting DIOR-balloon in porcine coronary arteries. Catheter Cardiovasc Interv. (2010) 76:395–403. 10.1002/ccd.22468 20839356

[27] Cortese B Bertoletti A. Paclitaxel coated balloons for coronary artery interventions: a comprehensive review of preclinical and clinical data. Int J Cardiol. (2012) 161:4–12. 10.1016/j.ijcard.2011.08.855 21955612

[28] Gruber P Braun C Kahles T Hlavica M Anon J Diepers M Percutaneous transluminal angioplasty using the novel drug-coated balloon catheter SeQuent Please NEO for the treatment of symptomatic intracranial severe stenosis: feasibility and safety study. J Neurointerv Surg. (2019) 11:719–22. 10.1136/neurintsurg-2018-014378 30415229

[29] Torii S Kolodgie FD Virmani R Finn AV. IN.PACT admiral drug-coated balloons in peripheral artery disease: current perspectives. Med Devices (Auckl). (2019) 12:53–64. 10.2147/MDER.S165620 30858737 PMC6385763

[30] Peterson S Hasenbank M Silvestro C Raina SIN. PACT Admiral drug-coated balloon: durable, consistent and safe treatment for femoropopliteal peripheral artery disease. Adv Drug Deliv Rev. (2017) 112:69–77. 10.1016/j.addr.2016.10.003 27771367

[31] Jordan MA Toso RJ Thrower D Wilson L. Mechanism of mitotic block and inhibition of cell proliferation by taxol at low concentrations. Proc Natl Acad Sci U S A. (1993) 90:9552–6. 10.1073/pnas.90.20.9552 8105478 PMC47607

[32] Yang CH Horwitz SB. Taxol((R)): the first microtubule stabilizing agent. Int J Mol Sci. (2017) 18:1733. 10.3390/ijms18081733 28792473 PMC5578123

[33] Li J Kim SG Blenis J. Rapamycin: one drug, many effects. Cell Metab. (2014) 19:373–9. 10.1016/j.cmet.2014.01.001 24508508 PMC3972801

[34] Palmerini T Benedetto U Biondi-Zoccai G Della Riva D Bacchi-Reggiani L Smits PC Long-term safety of drug-eluting and bare-metal stents: evidence from a comprehensive network meta-analysis. J Am Coll Cardiol. (2015) 65:2496–507. 10.1016/j.jacc.2015.04.017 26065988

[35] Marlevi D Edelman ER. Vascular lesion-specific drug delivery systems: JACC state-of-the-art review. J Am Coll Cardiol. (2021) 77:2413–31. 10.1016/j.jacc.2021.03.307 33985687 PMC8238531

[36] Scheller B. Opportunities and limitations of drug-coated balloons in interventional therapies. Herz. (2011) 36:232–9. 10.1007/s00059-011-3462-3 21541736

[37] Belkacemi A Agostoni P Nathoe HM Voskuil M Shao C Van Belle E First results of the DEB-AMI (drug eluting balloon in acute ST-segment elevation myocardial infarction) trial: a multicenter randomized comparison of drug-eluting balloon plus bare-metal stent versus bare-metal stent versus drug-eluting stent in primary percutaneous coronary intervention with 6-month angiographic, intravascular, functional, and clinical outcomes. J Am Coll Cardiol. (2012) 59:2327–37. 10.1016/j.jacc.2012.02.027 22503057

[38] Januszek R Bil J Gilis-Malinowska N Staszczak B Figatowski T Milewski M Long-term outcomes following drug-eluting balloon or thin-strut drug-eluting stents for treatment of in-stent restenosis stratified by duration of dual antiplatelet therapy (DEB-dragon registry). Postepy Kardiol Interwencyjnej. (2022) 18:14–26. 10.5114/aic.2022.115631 35982740 PMC9199027

[39] Laksono S Setianto B Surya SP. Drug-eluting balloon: is it useful? Egypt Heart J. (2020) 72:80. 10.1186/s43044-020-00116-7 33175218 PMC7658274

[40] Latib A Colombo A Castriota F Micari A Cremonesi A De Felice F A randomized multicenter study comparing a paclitaxel drug-eluting balloon with a paclitaxel-eluting stent in small coronary vessels: the BELLO (balloon elution and late loss optimization) study. J Am Coll Cardiol. (2012) 60:2473–80. 10.1016/j.jacc.2012.09.020 23158530

[41] Jeger RV Farah A Ohlow MA Mangner N Mobius-Winkler S Leibundgut G Drug-coated balloons for small coronary artery disease (BASKET-SMALL 2): an open-label randomised non-inferiority trial. Lancet. (2018) 392:849–56. 10.1016/S0140-6736(18)31719-7 30170854

[42] Cortese B Di Palma G Guimaraes MG Piraino D Orrego PS Buccheri D Drug-coated balloon versus drug-eluting stent for small coronary vessel disease: PICCOLETO II randomized clinical trial. JACC Cardiovasc Interv. (2020) 13:2840–49. 10.1016/j.jcin.2020.08.035 33248978

[43] Unverdorben M Kleber FX Heuer H Figulla HR Vallbracht C Leschke M Treatment of small coronary arteries with a paclitaxel-coated balloon catheter in the PEPCAD I study: are lesions clinically stable from 12 to 36 months? EuroIntervention. (2013) 9:620–8. 10.4244/EIJV9I5A99 24058078

[44] Mathey DG Wendig I Boxberger M Bonaventura K Kleber FX. Treatment of bifurcation lesions with a drug-eluting balloon: the PEPCAD V (paclitaxel eluting PTCA balloon in coronary artery disease) trial. EuroIntervention. (2011) 7(Suppl K):K61–5. 10.4244/EIJV7SKA11 22027730

[45] Kleber FX Rittger H Ludwig J Schulz A Mathey DG Boxberger M Drug eluting balloons as stand alone procedure for coronary bifurcational lesions: results of the randomized multicenter PEPCAD-BIF trial. Clin Res Cardiol. (2016) 105:613–21. 10.1007/s00392-015-0957-6 26768146

[46] Liu H Zhao Y Lu Y Zhou S Zhang Y Zhao J The drug coated balloon-only strategy for treatment of de novo left main coronary artery bifurcation lesion: stentless strategy. Clin Appl Thromb Hemost. (2022) 28:10760296221118489. 10.1177/10760296221118489 35945818 PMC9373168

[47] Jing QM Zhao X Han YL Gao LL Zheng Y Li ZQ A drug-eluting balloon for the treatment of coronary bifurcation lesions in the side branch: a prospective multicenter randomized (BEYOND) clinical trial in China. Chin Med J (Engl). (2020) 133:899–908. 10.1097/CM9.0000000000000743 32265425 PMC7176447

[48] Kitani S Igarashi Y Tsuchikane E Nakamura S Seino Y Habara M Efficacy of drug-coated balloon angioplasty after directional coronary atherectomy for coronary bifurcation lesions (DCA/DCB registry). Catheter Cardiovasc Interv. (2021) 97:E614–E623. 10.1002/ccd.29185 32776689

[49] Yu X Ji F Xu F Zhang W Wang X Lu D Efficacy of paclitaxel coated balloon in the treatment of primary coronary artery lesions with a diameter of 2.8 mm and above. Chin J Cardiovasc Dis. (2018) 46:32–8. 10.3760/cma.j.issn.0253-3758.2018.01.006 29374935

[50] Rosenberg M Waliszewski M Krackhardt F Chin K Wan Ahmad WA Caramanno G Drug coated balloon-only strategy in de novo lesions of large coronary vessels. J Interv Cardiol. (2019) 2019:6548696. 10.1155/2019/6548696 31772539 PMC6739788

[51] Lu W Zhu Y Han Z Sun G Qin X Wang Z Short-term outcomes from drug-coated balloon for coronary de novo lesions in large vessels. J Cardiol. (2019) 73:151–5. 10.1016/j.jjcc.2018.07.008 30366637

[52] Wei X Wang X Zhao T Lin Y Chen B, D. o. C. M., the Fifth Affiliated Hospital of Sun Yat sen University. Efficacy and safety of paclitaxel coated balloon in the treatment of primary coronary artery disease. Chin J Atherosclerosis. (2019) 27:150–5.

[53] Hu FW Chang S Li Q Zhu YX Wang XY Cheng YW Long-term clinical outcomes after percutaneous coronary intervention with drug-coated balloon-only strategy in de novo lesions of large coronary arteries. Front Cardiovasc Med. (2022) 9:882303. 10.3389/fcvm.2022.882303 35911516 PMC9329593

[54] Koln PJ Scheller B Liew HB Rissanen TT Ahmad WA Weser R Treatment of chronic total occlusions in native coronary arteries by drug-coated balloons without stenting—a feasibility and safety study. Int J Cardiol. (2016) 225:262–7. 10.1016/j.ijcard.2016.09.105 27741486

[55] Mitsis A Gragnano F. Myocardial infarction with and without ST-segment elevation: a contemporary reappraisal of similarities and differences. Curr Cardiol Rev. (2021) 17:e230421189013. 10.2174/1573403X16999201210195702 33305709 PMC8762150

[56] Keeley EC Boura JA Grines CL. Primary angioplasty versus intravenous thrombolytic therapy for acute myocardial infarction: a quantitative review of 23 randomised trials. Lancet. (2003) 361:13–20. 10.1016/S0140-6736(03)12113-7 12517460

[57] Sabate M. Drug-coated balloon for diabetic patients with small coronary vessels: is it the way to go? JACC Cardiovasc Interv. (2021) 14:1799–800. 10.1016/j.jcin.2021.07.011 34412798

[58] Wang Y Duan Y Su H Wang Y Bai W Chen H Acute non-ST segment elevation myocardial infarction as the first manifestation of Takayasu arteritis in a 16-year-old female patient: a case report and literature review. J Int Med Res. (2023) 51:3000605231178599. 10.1177/03000605231178599 37340716 PMC10288406

[59] Xu X Jin S Liu T. Drug-coated balloon angioplasty for coronary stenotic lesions in a paediatric patient after Kawasaki disease. Cardiol Young. (2022) 32:340–2. 10.1017/S104795112100295X 34429174

[60] Hirose M Narita J Hashimoto K Ishii R Ishida H Ozono K. Use of drug-coated balloon instead of drug-eluting stent for pediatric cardiac allograft vasculopathy. Ann Pediatr Cardiol. (2023) 16:45–7. 10.4103/apc.apc_47_22 37287837 PMC10243651

[61] Liistro F Angioli P Porto I Ducci K Falsini G Ventoruzzo G Drug-eluting balloon versus drug-eluting stent for complex femoropopliteal arterial lesions: the DRASTICO study. J Am Coll Cardiol. (2019) 74:205–15. 10.1016/j.jacc.2019.04.057 31296293

[62] Bausback Y Wittig T Schmidt A Zeller T Bosiers M Peeters P Drug-eluting stent versus drug-coated balloon revascularization in patients with femoropopliteal arterial disease. J Am Coll Cardiol. (2019) 73:667–9. 10.1016/j.jacc.2018.11.039 30765033

[63] Voiculescu A Grabensee B Jung G Modder U Sandmann W. Renovascular disease: a review of diagnostic and therapeutic procedures. Minerva Urol Nefrol. (2006) 58:127–49. 17124483

[64] Prati F Di Mario C Moussa I Reimers B Mallus MT Parma A In-stent neointimal proliferation correlates with the amount of residual plaque burden outside the stent: an intravascular ultrasound study. Circulation. (1999) 99:1011–4. 10.1161/01.cir.99.8.1011 10051293

[65] Takahashi EA McKusick MA Bjarnason H Piryani A Harmsen WS Misra S. Treatment of in-stent restenosis in patients with renal artery stenosis. J Vasc Interv Radiol. (2016) 27:1657–62. 10.1016/j.jvir.2016.05.041 27503035 PMC11520194

[66] Bi YH Ren JZ Yi MF Li JD Han XW. Drug coated balloon angioplasty for renal artery stenosis due to Takayasu arteritis: report of five cases. World J Clin Cases. (2019) 7:2888–93. 10.12998/wjcc.v7.i18.2888 31616707 PMC6789392

[67] Ngo AT Markar SR De Lijster MS Duncan N Taube D Hamady MS. A systematic review of outcomes following percutaneous transluminal angioplasty and stenting in the treatment of transplant renal artery stenosis. Cardiovasc Intervent Radiol. (2015) 38:1573–88. 10.1007/s00270-015-1134-z 26088719

[68] Ding M Feng N Tang D Feng J Li Z Jia M Melatonin prevents Drp1-mediated mitochondrial fission in diabetic hearts through SIRT1-PGC1alpha pathway. J Pineal Res. (2018) 65:e 12491. 10.1111/jpi.12491 PMC6099285 29575122

[69] Kozlova EV Bulkina OS Lopukhova VV Mironov VM Kushnir VV Vasilenko EI Management of the patient with renal artery fibromuscular dysplasia: clinical case. Kardiologiia. (2022) 62:65–8. 10.18087/cardio.2022.8.n2069 36066990

[70] Kirtane AJ Gupta A Iyengar S Moses JW Leon MB Applegate R Safety and efficacy of drug-eluting and bare metal stents: comprehensive meta-analysis of randomized trials and observational studies. Circulation. (2009) 119:3198–206. 10.1161/CIRCULATIONAHA.108.826479 19528338

[71] Lassen JF Holm NR Stankovic G Lefevre T Chieffo A Hildick-Smith D Percutaneous coronary intervention for coronary bifurcation disease: consensus from the first 10 years of the European bifurcation club meetings. EuroIntervention. (2014) 10:545–60. 10.4244/EIJV10I5A97 25256198

[72] Hildick-Smith D Arunothayaraj S Stankovic G Chen SL. Percutaneous coronary intervention of bifurcation lesions. EuroIntervention. (2022) 18:e273–91. 10.4244/EIJ-D-21-01065 35866256 PMC9912967

[73] Burzotta F Lassen JF Lefevre T Banning AP Chatzizisis YS Johnson TW Percutaneous coronary intervention for bifurcation coronary lesions: the 15(th) consensus document from the European bifurcation club. EuroIntervention. (2021) 16:1307–17. 10.4244/EIJ-D-20-00169 33074152 PMC8919527

[74] Piraino D Cortese B Buccheri D Dendramis G Andolina G. Healing after coronary artery dissection: the effect of a drug coated balloon angioplasty in a bifurcation lesion. A lesson from intravascular ultrasound analysis. Int J Cardiol. (2016) 203:298–300. 10.1016/j.ijcard.2015.10.156 26520278

[75] Piraino D Carella M Buccheri D Andolina G. Bifurcation lesions: bioresorbable vascular scaffold and drug coated balloon, an efficacy association. Lessons from optimal coherence tomography. Int J Cardiol. (2016) 212:92–3. 10.1016/j.ijcard.2016.03.048 27038711

[76] Yu X Ji FS Xu F Zhang WD Wang XY Lu D Therapeutic efficacy of paclitaxel-coated balloon for de novo coronary lesions with diameters larger than 2.8 mm. Zhonghua Xin Xue Guan Bing Za Zhi. (2018) 46:32–8. 10.3760/cma.j.issn.0253-3758.2018.01.006 29374935

[77] Galla JM Whitlow PL. Coronary chronic total occlusion. Cardiol Clin. (2010) 28:71–9. 10.1016/j.ccl.2009.10.003 19962050

[78] Yoneda K Takahashi T Kishi K. Over ten years’ follow-up of chronic total coronary occlusion angioplasty. Cardiovasc Revasc Med. (2021) 25:44–6. 10.1016/j.carrev.2020.10.013 33183984

[79] Wohrle J Werner GS. Paclitaxel-coated balloon with bare-metal stenting in patients with chronic total occlusions in native coronary arteries. Catheter Cardiovasc Interv. (2013) 81:793–9. 10.1002/ccd.24409 22511572

[80] Her AY Shin ES Bang LH Nuruddin AA Tang Q Hsieh IC Drug-coated balloon treatment in coronary artery disease: recommendations from an Asia-Pacific consensus group. Cardiol J. (2021) 28:136–49. 10.5603/CJ.a2019.0093 31565793 PMC8105061

[81] Ielasi A Buono A Pellicano M Tedeschi D Loffi M Donahue M A hybrid approach evaluating a drug-coated balloon in combination with a new-generation drug-eluting stent in the treatment of de novo diffuse coronary artery disease: the HYPER pilot study. Cardiovasc Revasc Med. (2021) 28:14–9. 10.1016/j.carrev.2020.07.036 32933874

[82] Gitto M Sticchi A Chiarito M Novelli L Leone PP Mincione G Drug-coated balloon angioplasty for de novo lesions on the left anterior descending artery. Circ Cardiovasc Interv. (2023) 16:e 013232. 10.1161/CIRCINTERVENTIONS.123.013232 37874646

[83] Scarsini R Fezzi S Leone AM De Maria GL Pighi M Marcoli M Functional patterns of coronary disease: diffuse, focal, and serial lesions. JACC Cardiovasc Interv. (2022) 15:2174–91. 10.1016/j.jcin.2022.07.015 36357022

[84] Ho HH Tan J Ooi YW Loh KK Aung TH Yin NT Preliminary experience with drug-coated balloon angioplasty in primary percutaneous coronary intervention. World J Cardiol. (2015) 7:311–4. 10.4330/wjc.v7.i6.311 26131335 PMC4478565

[85] Vos NS Fagel ND Amoroso G Herrman JR Patterson MS Piers LH Paclitaxel-coated balloon angioplasty versus drug-eluting stent in acute myocardial infarction: the revelation randomized trial. JACC Cardiovasc Interv. (2019) 12:1691–9. 10.1016/j.jcin.2019.04.016 31126887

[86] Scheller B Ohlow MA Ewen S Kische S Rudolph TK Clever YP Bare metal or drug-eluting stent versus drug-coated balloon in non-ST-elevation myocardial infarction: the randomised PEPCAD NSTEMI trial. EuroIntervention. (2020) 15:1527–33. 10.4244/EIJ-D-19-00723 31659986

[87] Rissanen TT Uskela S Siljander A Karkkainen JM Mantyla P Mustonen J Percutaneous coronary intervention of complex calcified lesions with drug-coated balloon after rotational atherectomy. J Interv Cardiol. (2017) 30:139–46. 10.1111/joic.12366 28116778

[88] Basavarajaiah S Latib A Shannon J Naganuma T Sticchi A Bertoldi L Drug-eluting balloon in the treatment of in-stent restenosis and diffuse coronary artery disease: real-world experience from our registry. J Interv Cardiol. (2014) 27:348–55. 10.1111/joic.12129 24815951

[89] Norgren L Hiatt WR Dormandy JA Nehler MR Harris KA Fowkes FG. Tasc II working group. inter-society consensus for the management of peripheral arterial disease (TASC II). J Vasc Surg. (2007) 45(Suppl S):S5–67. 10.1016/j.jvs.2006.12.037 17223489

[90] Stroke O E Tendera M Aboyans V Bartelink ML Baumgartner I Clement D ESC guidelines on the diagnosis and treatment of peripheral artery diseases: document covering atherosclerotic disease of extracranial carotid and vertebral, mesenteric, renal, upper and lower extremity arteries: the task force on the diagnosis and treatment of peripheral artery diseases of the European society of cardiology (ESC). Eur Heart J. (2011) 32:2851–906. 10.1093/eurheartj/ehr211 21873417

[91] Kayssi A Al-Atassi T Oreopoulos G Roche-Nagle G Tan KT Rajan DK. Drug-eluting balloon angioplasty versus uncoated balloon angioplasty for peripheral arterial disease of the lower limbs. Cochrane Database Syst Rev. (2016) 2016:CD011319. 10.1002/14651858.CD011319.pub2 27490003 PMC8504434

[92] Tepe G Laird J Schneider P Brodmann M Krishnan P Micari A Drug-coated balloon versus standard percutaneous transluminal angioplasty for the treatment of superficial femoral and popliteal peripheral artery disease: 12-month results from the IN.PACT SFA randomized trial. Circulation. (2015) 131:495–502. 10.1161/CIRCULATIONAHA.114.011004 25472980 PMC4323569

[93] Yoshida M Kimura A Katsuragi K Numano F Sasazuki T. DNA typing of HLA-B gene in Takayasu’s arteritis. Tissue Antigens. (1993) 42:87–90. 10.1111/j.1399-0039.1993.tb02242.x 7903491

[94] Yamamoto T Shirai K Okamura K Urata H. Two years efficacy of paclitaxel-coated balloon dilation for in-stent renal artery restenosis due to Takayasu arteritis. Am J Case Rep. (2019) 20:1089–93. 10.12659/AJCR.916105 31341156 PMC6676994

[95] Chhabra L Zain MA Siddiqui WJ. Angioplasty. In: StatPearls. Treasure Island (FL): StatPearls (2023).

[96] Auffret V Berland J Barragan P Waliszewski M Bonello L Delarche N Treatment of drug-eluting stents in-stent restenosis with paclitaxel-coated balloon angioplasty: insights from the French “real-world” prospective GARO registry. Int J Cardiol. (2016) 203:690–6. 10.1016/j.ijcard.2015.11.031 26583844

[97] Virga V Stabile E Biamino G Salemme L Cioppa A Giugliano G Drug-eluting balloons for the treatment of the superficial femoral artery in-stent restenosis: 2-year follow-up. JACC Cardiovasc Interv. (2014) 7:411–5. 10.1016/j.jcin.2013.11.020 24630884

[98] Samady H Mekonnen G. Drug-eluting balloons: effective and durable treatment for in-stent restenosis. JACC Cardiovasc Interv. (2013) 6:577–9. 10.1016/j.jcin.2013.04.002 23787232

[99] Alfonso F Perez-Vizcayno MJ Cardenas A Garcia Del Blanco B Seidelberger B Iniguez A A randomized comparison of drug-eluting balloon versus everolimus-eluting stent in patients with bare-metal stent-in-stent restenosis: the RIBS V Clinical Trial (Restenosis Intra-stent of Bare Metal Stents: paclitaxel-eluting balloon vs. everolimus-eluting stent). J Am Coll Cardiol. (2014) 63:1378–86. 10.1016/j.jacc.2013.12.006 24412457

[100] Giacoppo D Alfonso F Xu B Claessen B Adriaenssens T Jensen C Drug-coated balloon angioplasty versus drug-eluting stent implantation in patients with coronary stent restenosis. J Am Coll Cardiol. (2020) 75:2664–78. 10.1016/j.jacc.2020.04.006 32466881

[101] Wang G Zhao Q Chen Q Zhang X Tian L Zhang X. Comparison of drug-eluting balloon with repeat drug-eluting stent for recurrent drug-eluting stent in-stent restenosis. Coron Artery Dis. (2019) 30:473–80. 10.1097/MCA.0000000000000784 31464729 PMC6791562

[102] Wolny R Kowalik I Januszek R Bil J Figatowski T Milewski M Long-term outcomes following drug-eluting balloons vs. thin-strut drug-eluting stents for treatment of recurrent restenosis in drug-eluting stents. Kardiol Pol. (2022) 80:765–73. 10.33963/KP.a2022.0106 35445739

[103] Shoeib O Burzotta F. Recurrent restenosis in drug-eluting stents: still looking for the best treatment? Kardiol Pol. (2022) 80:738–40. 10.33963/KP.a2022.0146 35703432

[104] Tzafriri AR Vukmirovic N Kolachalama VB Astafieva I Edelman ER. Lesion complexity determines arterial drug distribution after local drug delivery. J Control Release. (2010) 142:332–8. 10.1016/j.jconrel.2009.11.007 19925836 PMC2994187

[105] Hwang CW Levin AD Jonas M Li PH Edelman ER. Thrombosis modulates arterial drug distribution for drug-eluting stents. Circulation. (2005) 111:1619–26. 10.1161/01.CIR.0000160363.30639.37 15795325

[106] Speck U Hackel A Schellenberger E Kamann S Lochel M Clever YP Drug distribution and basic pharmacology of paclitaxel/resveratrol-coated balloon catheters. Cardiovasc Intervent Radiol. (2018) 41:1599–1610. 10.1007/s00270-018-2018-9 29968090 PMC6132862

[107] Selvarani R Mohammed S Richardson A. Effect of rapamycin on aging and age-related diseases-past and future. Geroscience. (2021) 43:1135–58. 10.1007/s11357-020-00274-1 33037985 PMC8190242

[108] Weaver BA. How taxol/paclitaxel kills cancer cells. Mol Biol Cell. (2014) 25:2677–81. 10.1091/mbc.E14-04-0916 25213191 PMC4161504

[109] Liebmann JE Cook JA Lipschultz C Teague D Fisher J Mitchell JB. Cytotoxic studies of paclitaxel (Taxol) in human tumour cell lines. Br J Cancer. (1993) 68:1104–9. 10.1038/bjc.1993.488 7903152 PMC1968657

[110] Wessely R Kastrati A Mehilli J Dibra A Pache J Schomig A. Randomized trial of rapamycin- and paclitaxel-eluting stents with identical biodegradable polymeric coating and design. Eur Heart J. (2007) 28:2720–5. 10.1093/eurheartj/ehm425 17921531

[111] Alfonso F Perez-Vizcayno MJ Cardenas A Garcia del Blanco B Garcia-Touchard A Lopez-Minguez JR A prospective randomized trial of drug-eluting balloons versus everolimus-eluting stents in patients with in-stent restenosis of drug-eluting stents: the RIBS IV randomized clinical trial. J Am Coll Cardiol. (2015) 66:23–33. 10.1016/j.jacc.2015.04.063 26139054

[112] Granada JF Stenoien M Buszman PP Tellez A Langanki D Kaluza GL Mechanisms of tissue uptake and retention of paclitaxel-coated balloons: impact on neointimal proliferation and healing. Open Heart. (2014) 1:e 000117. 10.1136/openhrt-2014-000117 PMC4189287 25332821

[113] Werk M Albrecht T Meyer DR Ahmed MN Behne A Dietz U Paclitaxel-coated balloons reduce restenosis after femoro-popliteal angioplasty: evidence from the randomized PACIFIER trial. Circ Cardiovasc Interv. (2012) 5:831–40. 10.1161/CIRCINTERVENTIONS.112.971630 23192918

[114] Stolzenburg N Breinl J Bienek S Jaguszewski M Lochel M Taupitz M Paclitaxel-Coated balloons: investigation of drug transfer in healthy and atherosclerotic arteries—first experimental results in rabbits at low inflation pressure. Cardiovasc Drugs Ther. (2016) 30:263–70. 10.1007/s10557-016-6658-1 27033233 PMC4919377

[115] Holmes DR Vlietstra RE Smith HC Vetrovec GW Kent KM Cowley MJ Restenosis after percutaneous transluminal coronary angioplasty (PTCA): a report from the PTCA registry of the National Heart, Lung, and Blood Institute. Am J Cardiol. (1984) 53:77C–81C. 10.1016/0002-9149(84)90752-5 6233894

[116] Kolachalama VB Pacetti SD Franses JW Stankus JJ Zhao HQ Shazly T Mechanisms of tissue uptake and retention in zotarolimus-coated balloon therapy. Circulation. (2013) 127:2047–55. 10.1161/CIRCULATIONAHA.113.002051 23584359 PMC3748613

[117] Ali RM Abdul Kader M Wan Ahmad WA Ong TK Liew HB Omar AF Treatment of coronary drug-eluting stent restenosis by a sirolimus- or paclitaxel-coated balloon. JACC Cardiovasc Interv. (2019) 12:558–66. 10.1016/j.jcin.2018.11.040 30898253

[118] Kelsch B Scheller B Biedermann M Clever YP Schaffner S Mahnkopf D Dose response to paclitaxel-coated balloon catheters in the porcine coronary overstretch and stent implantation model. Invest Radiol. (2011) 46:255–63. 10.1097/RLI.0b013e31820577df 21285890

[119] Gongora CA Shibuya M Wessler JD McGregor J Tellez A Cheng Y Impact of paclitaxel dose on tissue pharmacokinetics and vascular healing: a comparative drug-coated balloon study in the familial hypercholesterolemic swine model of superficial femoral in-stent restenosis. JACC Cardiovasc Interv. (2015) 8:1115–23. 10.1016/j.jcin.2015.03.020 26117470

[120] Plass CA Sabdyusheva-Litschauer I Bernhart A Samaha E Petnehazy O Szentirmai E Time course of endothelium-dependent and -independent coronary vasomotor response to coronary balloons and stents. Comparison of plain and drug-eluting balloons and stents. JACC Cardiovasc Interv. (2012) 5:741–51. 10.1016/j.jcin.2012.03.021 22814779

[121] Nakamura T Brott BC Brants I Panchal D Li J Chen JP Vasomotor function after paclitaxel-coated balloon post-dilation in porcine coronary stent model. JACC Cardiovasc Interv. (2011) 4:247–55. 10.1016/j.jcin.2010.08.028 21349465

[122] Schneider PA Giasolli R Ebner A Virmani R Granada JF. Early experimental and clinical experience with a focal implant for lower extremity post-angioplasty dissection. JACC Cardiovasc Interv. (2015) 8:347–54. 10.1016/j.jcin.2014.07.032 25700758

[123] Sharma S Raut N Potdar A. Spontaneous coronary artery dissection: case series and review of literature. Indian Heart J. (2016) 68:480–5. 10.1016/j.ihj.2015.11.039 27543469 PMC4990736

[124] Tepe G Zeller T Schnorr B Claussen CD Beschorner U Brechtel K High-grade, non-flow-limiting dissections do not negatively impact long-term outcome after paclitaxel-coated balloon angioplasty: an additional analysis from the THUNDER study. J Endovasc Ther. (2013) 20:792–800. 10.1583/13-4392R.1 24325695

[125] Wurdinger M Cammann VL Ghadri JR Templin C. Spontaneous coronary artery dissection: a rare event? Heart Fail Clin. (2022) 18:189–99. 10.1016/j.hfc.2021.07.015 34776079

[126] Fanelli F Cannavale A Gazzetti M D’Adamo A. Commentary: how do we deal with dissection after angioplasty? J Endovasc Ther. (2013) 20:801–4. 10.1583/13-4392C.1 24325696

[127] Voute MT Stathis A Schneider PA Thomas SD Brodmann M Armstrong EJ Delphi consensus study toward a comprehensive classification system for angioplasty-induced femoropopliteal dissection: the DISFORM study. JACC Cardiovasc Interv. (2021) 14:2391–401. 10.1016/j.jcin.2021.07.056 34736739

[128] Gray WA Cardenas JA Brodmann M Werner M Bernardo NI George JC Treating post-angioplasty dissection in the femoropopliteal arteries using the tack endovascular system: 12-month results from the TOBA II study. JACC Cardiovasc Interv. (2019) 12:2375–84. 10.1016/j.jcin.2019.08.005 31806218

[129] Kobayashi N Hirano K Yamawaki M Araki M Sakai T Sakamoto Y Simple classification and clinical outcomes of angiographic dissection after balloon angioplasty for femoropopliteal disease. J Vasc Surg. (2018) 67:1151–58. 10.1016/j.jvs.2017.08.092 29242063

[130] Kastrati A Schomig A Dietz R Neumann FJ Richardt G. Time course of restenosis during the first year after emergency coronary stenting. Circulation. (1993) 87:1498–505. 10.1161/01.cir.87.5.1498 8491004

[131] Gao L Wang YB Jing J Zhang M Chen YD. Drug-eluting balloons versus new generation drug-eluting stents for the management of in-stent restenosis: an updated meta-analysis of randomized studies. J Geriatr Cardiol. (2019) 16:448–57. 10.11909/j.issn.1671-5411.2019.06.002 31308837 PMC6612611

[132] Ngam PI Ong CC Chai P Wong SS Liang CR Teo LLS. Computed tomography coronary angiography—past, present and future. Singapore Med J. (2020) 61:109–15. 10.11622/smedj.2020028 32488269 PMC7905109

[133] Hui L Shin ES Jun EJ Bhak Y Garg S Kim TH Impact of dissection after drug-coated balloon treatment of de novo coronary lesions: angiographic and clinical outcomes. Yonsei Med J. (2020) 61:1004–12. 10.3349/ymj.2020.61.12.1004 33251774 PMC7700881

[134] Tahara S Chamie D Baibars M Alraies C Costa M. Optical coherence tomography endpoints in stent clinical investigations: strut coverage. Int J Cardiovasc Imaging. (2011) 27:271–87. 10.1007/s10554-011-9796-3 21394615 PMC4459645

[135] Tanigawa J Barlis P Di Mario C. Intravascular optical coherence tomography: optimisation of image acquisition and quantitative assessment of stent strut apposition. EuroIntervention. (2007) 3:128–36. 19737696

[136] Ghekiere O Salgado R Buls N Leiner T Mancini I Vanhoenacker P Image quality in coronary CT angiography: challenges and technical solutions. Br J Radiol. (2017) 90:20160567. 10.1259/bjr.20160567 28055253 PMC5605061

